# Fresh Shelves, Healthy Pantries: A Pilot Intervention Trial in Baltimore City Food Pantries

**DOI:** 10.3390/ijerph192315740

**Published:** 2022-11-26

**Authors:** Bengucan Gunen, Melissa M. Reznar, Sally Yan, Lisa Poirier, Nathan Katragadda, Shahmir H. Ali, Samantha M. Sundermeir, Joel Gittelsohn

**Affiliations:** 1 Human Nutrition, Department of International Health, Johns Hopkins Bloomberg School of Public Health, Baltimore, MD 21205, USA; 2 School of Health Sciences, Oakland University, Rochester, MI 48309, USA

**Keywords:** food pantry, food stocking, food acquisition, food assortment scoring tool, food insecurity

## Abstract

The objective of this study was to evaluate the impact of a multi-modal pilot intervention on the stocking and acquisition of healthy foods in urban food pantries. An intervention that consisted of three 8-week phases, each focused on promotion of one food group: (1) lean & low-sodium proteins; (2) fruits & vegetables; and (3) healthy carbohydrates was conducted in 3 intervention and 4 comparison food pantries. Food stocking variety scores measured changes in the stocking of promoted healthful foods at pantries. Food Assortment Scoring Tool (FAST) scores measured healthfulness of client bags. Intervention and comparison pantries showed an increase during the study in the total variety score for promoted options, with no significant differences between groups. Mean healthfulness scores for intervention client bags (n = 34) significantly increased from 58.2 to 74.9 (*p* < 0.001). This pilot trial identified logistically feasible strategies to promote healthy options effectively in food pantries, even in pantries with limited resources.

## 1. Introduction

Food pantries are locations that distribute food at no cost to those in need throughout the United States, with approximately 47 million people obtaining food from pantries every year [1,2]. Those that do visit pantries tend to be lower-income, have higher rates of unemployment than the general population, and visit pantries an average of 1.8 times per month [1,2]. However, the diets of food pantry clients tend to be of low quality, with inadequate intake of fruits, vegetables, and dairy [3]. This may be partly tied to the fact that financially challenged food pantries have difficulties stocking healthier food options [4,5]. Many pantries have limited inventory due to lack of funds to purchase items from their partnering food banks and lack resources such as transportation vehicles, storage, refrigeration, and volunteer help to procure food from additional sources [4,6,7,8]. Pantries with the capacity to receive a large amount of food from their partnering food banks and to service a greater number of clients typically have a healthier selection of foods [9]. However, even when healthier options are available, pantries may not display food items in ways that actively encourage selection of these options [10,11]. Interventions that seek to improve healthfulness of inventory and maximize placement of those items to promote consumption among clients, in addition to providing direct education (i.e., multilevel, multimodal interventions), are important in enabling food pantry clients to select healthier options [11,12].

Food pantry-based interventions have reported mixed results in improving client food selections or dietary consumption. One systematic review found that all twelve included interventions improved diet-related outcomes like food choice and diet quality [13], but another review found that the included studies did not promote sustained dietary change [14]. Given that food choice and eating behaviors differ because of both individual and environmental influences, experts suggest that dietary interventions that include both individual education as well as environmental and policy changes optimize effectiveness of behavior change [15,16,17,18]. Most food pantry interventions, however, have used only 1 mode of intervention (e.g., nutrition education [19], environmental improvements [11,20], or policy changes) [3,21]. Few intervention studies have combined multi-modal approaches to both increase healthier food options stocked at pantries and increase their selection among food pantry users.

To address this gap, we developed and implemented a multi-component environmental, educational, and policy intervention in Baltimore City urban food pantries of different sizes to improve access and uptake of healthful foods by low-income families. The intervention targets multiple levels of the social ecological model [22] combined with key constructs of social cognitive theory geared toward behavior change [23] to evaluate whether targeting multiple components impacts the healthfulness of the foods distributed at food pantries. The research questions in this study were: (1) Did the intervention strategies lead to increased variety of healthful foods stocked in intervention pantries compared to control pantries? (2) Does the use of environmental, educational and policy intervention strategies increase client selection of healthier options at intervention food pantries compared to control pantries? At the household- and individual-levels, we hypothesized that intervention food pantry clients will select healthier foods, as compared to comparison pantry clients. At the food pantry level, we hypothesized that intervention food pantries will increase the variety of healthful foods they stock.

## 2. Materials and Methods

### 2.1. Study Design

The Fresh Shelves, Healthy Pantries (FSHP) pilot intervention was implemented in 3 phases lasting 8 weeks each, between November 2018–October 2019. A phased approach was used given that the successful implementation of one mode of intervention would depend on the implementation of the other modes of intervention. Each of the 3 phases promoted foods within 1 of 3 food groups: (1) lean & low-sodium proteins; (2) fruits & vegetables; and (3) healthy carbohydrates. Interventionists (undergraduate and graduate students at Johns Hopkins University, trained in human subjects research, intervention strategies and dietary guidelines) worked with Maryland Food Bank’s (MFB) nutritionist to create food promotion stocking guidelines. These stocking guidelines specified foods to promote within each food group, ensuring that the promoted foods were: (a) culturally appropriate for the population served at food pantries, (b) lower in added sugar, saturated fat, and sodium, and (c) already available through MFB’s distribution system. At the time of the study, most food pantries in Baltimore City stocked canned legumes and vegetables, and frozen meals high in sodium; meats and dairy products high in saturated fat; canned fruits, breakfast cereal, baked goods, baking mixes, and beverages with added sugar [24]. The food groups and individual foods promoted in the intervention were selected based on healthful food items that were typically unavailable in Baltimore food pantries [24]. 

For the first phase, promotion of lean and low-sodium proteins included lean fresh and frozen meats such as skinless chicken breast and >85% lean ground beef, low-sodium canned legumes, dried legumes, and low-fat dairy products. Stocking guidelines indicated that managers should stock items with less than 10% of the daily value (DV) for saturated fats and sodium per serving. For the second phase, the promoted fruits and vegetables were fresh and unsweetened frozen fruits, unseasoned fresh and frozen vegetables with no sauces, canned fruits in 100% juice, and low-sodium canned vegetables. Managers were asked to order items with less than 10% of the DV for added sugars and sodium per serving. For the third phase, the healthy carbohydrates included unsweetened beverages, whole grains, and low-sugar breakfast cereals. Items with at least 10% of the DV for fiber, and at most 10% of the DV for added sugars were promoted. See Table 1. 

This study was reviewed by the Johns Hopkins Bloomberg School of Public Health Institutional Review Board and was deemed exempt.

### 2.2. Participants and Recruitment

Pantries in the MFB database of community-based networks in Baltimore City (n = 102) were stratified into tertiles of size, based on pounds of food distributed in the previous fiscal year: small (65 to 10,000 pounds), medium (10,001 to 24,600 pounds), and large (24,601 or more pounds). This stratification allowed the control and intervention pantries to be comparable in terms of resources and service capacity, while providing a variety of food pantries included in the trial. Exclusion criteria for food pantries included: (1) operating less than 1 day per week, (2) being located in a school (due to the different operational guidelines of food providers in the school setting), (3) already receiving nutrition education programming from the MFB or the University of Maryland Extension, and/or (4) having a new manager (<2 months in position). 

Interventionists attempted to contact the 102 food pantries at least 3 times, via telephone or e-mail, using contact information provided by the MFB. Of these, 21 food pantries could not be reached, and 44 were not eligible. Two small, 2 medium, and 3 large pantries were randomly selected out of 14 eligible and interested food pantries. Pantries were randomly assigned to intervention (1 small, 1 medium, 1 large) or comparison (1 small, 1 medium, 2 large) groups before consent discussions and baseline data collection. The number of food pantries was determined based on the feasibility of intervention delivery and the financial resources allocated for this study.

Researchers approached 10–12 clients from each pantry after clients received their food at baseline, after Phase 2 (mid-study), and after Phase 3 (post-intervention), and thus different individuals were recruited at each time point. Baseline data collection occurred in October/November 2018, mid-study data collection in May/June 2019, and post-intervention data collection in October/November 2019. Participants were recruited using a convenience sample of the clients who were present for food distribution at the time of data collection. Eligibility and oral consent discussions occurred at this point. To be eligible, clients had to be older than 18, fluently speak English, and have received food from the pantry that day. Participants’ names and contact information were not collected, and clients did not receive follow-ups throughout the study duration. Each participant received $10 gift cards as compensation for their time.

### 2.3. Study Procedures

Within each phase, a combination of multi-modal change strategies focusing on the food group for the phase was implemented, including policy, staff education and engagement, and client education and client-targeted environmental changes (see Table 1). 

#### 2.3.1. Policy Changes

Policy changes at pantries included increasing the supply and variety of promoted foods identified in the stocking guidelines and switching to a client choice distribution method, rather than the traditional prepackaged bags method. There was no written agreement to change stocking guidelines or to indefinitely implement client choice food distribution at intervention pantries; however, interventionists were present when managers placed orders via the MFB online menu and made suggestions on which items to order, to monitor compliance with the stocking guidelines. 

Intervention food pantries received an introductory incentive in the form of their choice of shelving unit, freezer, or refrigerator worth $300 to facilitate the stocking of promoted foods. During phase 3, interventionists utilized the Client Choice Pantry Handbook developed by the Akron-Canton Regional Foodbank [25] to discuss with food pantry managers which type of client choice food distribution would be ideal for each food pantry, considering each organization’s space, volunteer, and storage limitations. 

#### 2.3.2. Staff Education and Engagement

Interventionists provided nutrition education to pantry staff, informed them about stocking guidelines, provided print materials in the form of educational displays and brochures, and assisted with food distribution to facilitate the switch to client choice. Pantry staff participated in brief quizzes after each educational lesson to reinforce lesson content. In addition, food pantry staff received print materials outlining how to utilize the promoted healthy options at home, to be shared with clients during food distribution.

#### 2.3.3. Client Education and Environmental Change

Interventionists conducted nutrition educational sessions for clients. In addition, interventionists organized taste testing and cooking demonstrations featuring healthy foods within each of the intervention phases [25]. 

Interventionists also used print materials—flyers, recipe cards, posters—as well as social media to engage clients. Other changes in the pantry environment targeted to clients included rearranging shelves to prominently display healthy options at eye-level relative to less healthy options and putting shelf tags near those promoted foods to draw attention to them. 

At study completion, control pantries (1 small, 1 medium, 2 large) were given a final report on intervention strategies and print materials.

### 2.4. Instruments

#### 2.4.1. Food Pantry Environmental Checklist (FPEC)

This instrument was developed based on in-depth interviews with food pantry managers and structured observations of the pantry environments in Baltimore City [24]. The FPEC was assessed for face validity by executives at the MFB and was used to collect pantry-level impact on the variety of healthful foods offered. Interventions completed the FPEC at baseline (October/November 2018) and after each of the 3 phases, (post-phase 1 February 2019, post-phase 2 [mid-study] May/June 2019, post-phase 3 October/November 2019). The FPEC consisted of 2 parts. The first part collected pantry-level information on the pantry’s sources of food, food distribution method, number of clients served, frequency of providing additional services to clients such as nutrition education and nudging of healthy options, guidelines for food ordering and distribution, volunteer capacity, hours of operation, and storage capacity. The second part of FPEC recorded the number of varieties of healthy foods stocked and promoted during each phase of the study, namely, the number of varieties for lean and low-sodium proteins, fruit and vegetable products low in added sugars and sodium, and healthy carbohydrates low in added sugars. 

#### 2.4.2. Client Questionnaire

This instrument was developed by the research team, in collaboration with the Lerner Center for Health Promotion, and the MFB’s Director of Nutrition [26]. The questionnaire was pilot tested with 10 food pantry clients from a food pantry not participating in the Fresh Shelves, Health Pantries trial. The questionnaire was designed to be interviewer-administered. Client-level data was collected at baseline, after Phase 2 (mid-study), and after Phase 3 (post-intervention). Collected data included sociodemographic information as well as the weight and varieties of types of food and food groups in each client’s bag at check out. Interventionists separated the contents of each participating client’s bag into food groups. Then, the overall weights of each food group were measured using a scale.

#### 2.4.3. Interventionist Form

Structured observations of food pantry environments, quiz scores of pantry managers at the conclusion of nutrition education sessions, and conduct of intervention activities, including cooking demonstrations and taste tests, were collected at each intervention visit by interventionists using REDCap, a secure web-based application that enables onsite research data collection. This information was used to inform the interpretation of study results.

### 2.5. Measures

#### 2.5.1. Food Stocking Variety Scores

Data from the FPEC was used to count the number of varieties of each promoted product category at baseline and after each phase. Stocking scores were calculated by summing the number of stocked varieties of promoted food groups. To assess the changes in the stocking of healthful foods at the pantry level by phase, variety change scores for each food group promoted during the intervention were calculated.

#### 2.5.2. Food Assortment Scoring Tool (FAST)

Healthfulness of client bags was calculated using FAST methodology, which has been validated to accurately assess the healthfulness of food selections at the food pantry setting and involves multiplying gross weight of 13 different food categories, relative to the weight of the whole bag, by a healthfulness factor and summing those weights [27]. FAST scores range from 0–100, and a higher score indicates a healthier client bag. 

### 2.6. Data Analysis

Descriptive statistics, mean ± standard deviation or percentages, and chi-square tests were calculated for pantry-level variety scores, client-level sociodemographics, and client-level FAST scores. Pearson’s Chi-squared test with Yates’ continuity correction were used for categorical variables, and Wilcoxon rank sum tests were used for continuous variables.

FAST scores were analyzed for normality using the Shapiro–Wilk normality test. Upon review of normality tests and histograms of FAST score distribution, we determined that non-parametric tests for FAST scores would be most appropriate. Wilcoxon rank sum tests were used to determine statistically significant changes in FAST scores of client bags between the intervention and comparison groups at the 3 time points of data collection and to assess group differences between time points.

Data were analyzed using R software (R version 3.5.3, R Foundation for Statistical Computing, Vienna, Austria, https://www.r-project.org/ (accessed on 1 February 2020)). Differences between treatment groups were statistically significant at *p* < 0.05.

## 3. Results

### 3.1. Food Stocking Variety (Research Question 1) 

The characteristics of the 7 participating food pantries at baseline data collection is shown in Table 2. In sum, although the 3 intervention and 4 comparison food pantries were similar in their hours of operation, comparison food pantries were more likely to supplement their food offerings with foods donated from venues besides MFB. On average, comparison food pantries also had more than twice as many hours of volunteer help, served twice as many clients, and distributed twice as much food, compared to intervention food pantries. 

**Figure 1:**
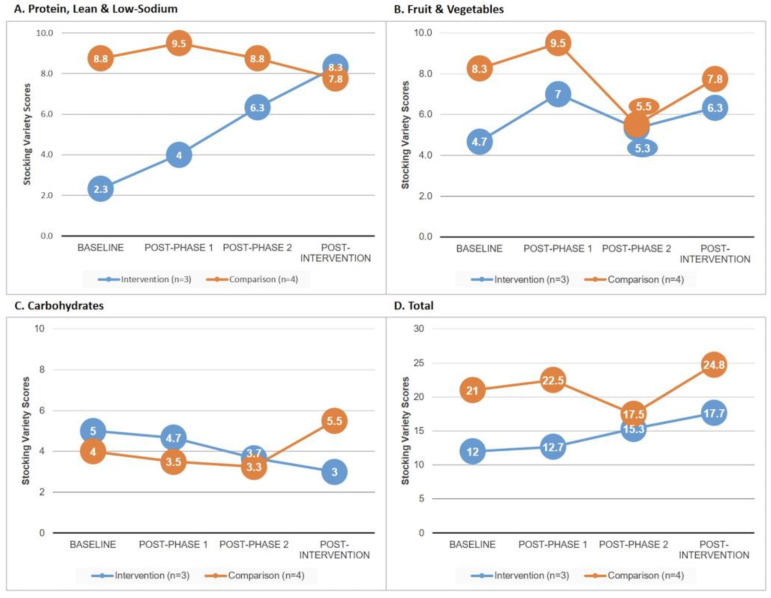
Stocking Variety Scores from baseline to post-intervention among intervention and comparison food pantries.

We did not see significant increases in the stocked varieties of promoted healthy options by treatment status within food group (Figure 1). For lean & low-sodium proteins, the difference in variety scores between post-intervention and baseline was +6.00 ± 5.29 for intervention pantries, and −1.00 ± 5.03 for comparison pantries (*p* = 0.13) (Figure 1A). For fruits & vegetables, the difference in variety scores between post-intervention and baseline was +1.67 ± 7.51 for intervention pantries, and +3.25 ± 7.46 for comparison pantries (*p* = 0.80) (Figure 1B). For healthy carbohydrates, the difference in variety scores between post-intervention and baseline was +5.33 ± 9.50 for intervention pantries, and +1.75 ± 6.50 for comparison pantries (*p* = 0.61) (Figure 1C). Overall, for all food groups combined, comparison food pantries stocked a greater variety of healthy foods from each food group than intervention pantries at each time point (Figure 1D). There were no significant differences between intervention and comparison pantry stocking variety scores, with both increasing overall during the course of the study.

### 3.2. Healthfulness of Client Bags: FAST Scores (Research Question 2)

The sociodemographic characteristics of participating food pantry clients are displayed in Table 3. Overall, a total of 135 clients in intervention pantries and 129 clients in comparison pantries participated in the study. We did not observe any statistically significant differences between the control and intervention group participants at any point for the following characteristics: household size, number of children in the household, race and ethnicity, WIC recipients. Comparison group participants were significantly younger than intervention group participants at all data collection points (*p* < 0.05).

The FSHP intervention was associated with significant differences in client FAST scores (Table 4). The average FAST score for intervention pantry clients was higher by an average of 16.4 points (*p* < 0.05), while average FAST score for clients of control pantries were lower by about 5 points over the course of the intervention (*p* < 0.05). 

## 4. Discussion

The Fresh Shelves Healthy Pantries (FSHP) pilot intervention utilized a comprehensive combination of policy, educational, and environmental strategies to increase selection of healthier items in pantries of various sizes and with diverse levels of resources. Our pilot trial incorporated several logistically feasible intervention strategies to promote healthy options in the food pantry setting. The food pantries participating in this study varied vastly in their capacity and food offerings, and comparison food pantries distributed a much larger volume of food at baseline. Furthermore, comparison food pantries were already stocking a greater variety of healthful options before the intervention. This relationship between food pantry “size” (i.e., amount of food received from a food bank and distributed to clients) and healthfulness of food offerings has been noted in previous literature [9]. Furthermore, stocks of healthier foods, like the lean proteins, fruits and vegetables, and carbohydrates featured in this study, would be more diverse if perishable versions (i.e., raw and unprocessed) of the foods within these categories were provided by pantries [28]. Although we provided offered refrigerators as an incentive in this study, the refrigerators may not be used for these specific foods, and a review study indicates that supply of healthier, perishable foods to food banks can be unpredictable [28].

Overall healthy food selections by food pantry intervention clients were higher at later time points than earlier time points. The pantries participating in this study were diverse in their organization capacity, similar to the food pantry landscape in the United States [29]. Previous multi-modal interventions have been effective at improving the supply and demand for healthy foods at food pantries in other settings; however, these have not incorporated nutrition education and have only recruited pantries already following the client choice model before the intervention [11]. 

The average healthfulness (FAST scores) of client food selections at intervention food pantries was higher after intervention time points, whereas the healthfulness of client food selections of comparison food pantries was somewhat stable. The positive difference in healthfulness scores at intervention pantries was attributed to an increased gross weight of whole grains, vegetable proteins, and processed fruits and vegetables. Based on food-pantry level FPEC data, we did not observe an increase in the stocking levels of whole grains and processed fruits and vegetables at the intervention food pantries. According to the field notes of interventionists, the stocking levels at food pantries were limited by structural barriers, such as the availability of storage space and the availability of promoted items on the MFB marketplace, and that the nutrition education component of the intervention nudged clients to choose a greater amount of the promoted items when they were available. This is in line with the findings from a systematic review of previous food pantry intervention studies, which have found nutrition education and environmental changes to be effective [13].

Our intervention was not without limitations. This pilot study included pantry- and client-level data from 7 pantries, although the client-level data included different individuals at each time point. It is possible that the findings would not be applicable to all food pantries in or outside of Baltimore city. We excluded food pantries in Baltimore City Public Schools due to the different operational guidelines of pantries in the school setting. In addition, we excluded pantries that operated less than 1 day a week. Individuals with limited English language skills were excluded from this study due to resource limitations, although they constitute an important sub-section of the food insecure population in the United States [30,31]. The FPEC did not evaluate the proportional amount (in weight or volume) of healthy versus unhealthy options. Food pantry clients were not followed up longitudinally, as many clients utilize their pantry only once or twice per month. The lack of longitudinal data collection resulted in analysis of group level differences at pantries rather than change scores among individuals. Another limitation is that this study aimed to test the feasibility of a multimodal food pantry-based intervention, and the sample size was not powered to provide statistical evidence on the impact of the intervention on food pantry- and client-level outcomes. As a result, we did not statistically control for differences in the sociodemographic composition of client groups, as we believe doing so would not provide statistically sound results. Further, we do not know if the differences in the healthfulness of client bags in intervention pantries were associated with a healthier overall diet for these clients, as our client-level data collection does not include dietary assessments. The long-term sustainability of this intervention cannot be determined, and should be considered in future work. For instance, future studies should evaluate the proportion of healthier foods selected by clients that is consumed and whether healthier selections continue to be selected during repeated visits by clients after the promotion of those products has concluded. Identifying which intervention component or combination of components are most effective should also be considered in future trials. Our observations from Baltimore City are supported by evidence on food pantries in other urban settings in the United States [4,5].

This intervention provides evidence for the feasibility of a multi-modal intervention at smaller, urban community-based food assistance organizations, and demonstrates a partnership between regional food banks, local food pantries and an academic institution to design and implement a nutrition intervention. Since the conclusion of this pilot trial, Baltimore City food pantries and the MFB shifted their operational strategies to increase the number of clients they served during the COVID-19 pandemic, while maintaining social distance. With the client choice food distribution method becoming less favorable, digital technologies at the food pantry setting may improve the sustainability and the scalability of environmental, educational and policy strategies found to be feasible in this pilot trial, while making emergency response safer and swifter [32,33]. Digital strategies such as videos, discussion forums, blog posts, recipes, and online flyers might be more sustainable and scalable for this setting, as most food pantry managers are technologically savvy, and submit orders and reports through the MFB website. At the time of this intervention, the MFB online ordering software did not allow food pantry managers to communicate which items they would like to order at a larger amount than what was currently available. Based on information recorded by interventionists, we observed that this feature was desired by food pantry managers. A real-time digital dashboard reflecting the most commonly ordered items could help the MFB staff demand more of these items from their retail partners or purchase these foods from manufacturers at a faster rate [34]. Interventionists also observed that pantry managers typically utilize text messaging to reach their clients, as opposed to social media. Although text messaging does not allow for pantries to reach new, potential clients, it might be an acceptable medium to provide nutrition education to regular clients.

## 5. Conclusions

This study provides evidence that a multi-modal approach of policy, educational and environmental change strategies are feasible in food pantries with varying levels of organizational capacity. Pantries in urban settings throughout the United States have the potential to benefit from these findings, as pantries in other cities face similar barriers to stocking and promoting healthy foods. We plan to carry out this intervention in a larger number of pantries and a larger total number of clients, which would improve the baseline comparability of pantries and thus improve stocking and selection of healthier foods. Taken together, food pantry interventions that incorporate nutrition education, healthier stocking policies, client-choice selection, and pantry environmental changes have the potential to crucially improve the nutritional security of food pantry clients.

## Figures and Tables

**Table 1:** Fresh Shelves, Healthy Pantries Intervention Phases.

Intervention Phase (Focus)	Food Pantry Staff Capacity Building (In-Person Training)	Educational/ Environmental Strategies	Policy
1: Lean, Low-Sodium Proteins	Benefits of lean, low-sodium proteins; procurement; nudging strategies; product placement.	Posters; Healthy alternatives displayed at eye-level near the entrance and posters.	Minimum depth-of-stock (minimum amount and variety per client).
2: Fresh, Frozen and Canned Produce	Benefits of produce; food safety; prevention of waste; client education; positive messaging.	Recipe cards with tips on use in daily cooking; Placement at the entrance.	Minimum depth-of stock (minimum amount and variety per client).
3: Healthy Carbohydrates	Consequences of high sugar consumption; benefits of whole grains; communication strategies for donors; community outreach.	Stoplight shelf labels for sugar and fiber content; Posters promoting low-sugar and whole grain items.	Restriction of high-sugar food and beverages (>10 g/serving) in procurement and stocking

**Table 2:** Baseline Characteristics of Participating Food Pantries.

Characteristic	Intervention (n = 3)	Comparison (n = 4)
Weight of food distributed annually (lbs), mean ± SD	32,558.33 ± 43,264.34	60,114.25 ± 69,936.90
Food sources, %		
Maryland Food Bank	71	43
Temporary Emergency Food Assistance Program (TEFAP)	14	19
Partner churches	0	24
Private donations	12	6
Other (incl. food retailers, wholesalers)	3	8
Number of clients served in past 2 weeks, mean ± SD	52.00 ± 30.00	126.00 ± 79.00
Number of volunteer hours in past 2 weeks, mean ± SD	50.00 ± 36.06	107.50 ± 99.15
Number of hours of weekly food distribution, mean ± SD	6.33 ± 2.08	6.00 ± 4.32

**Table 3:** Sociodemographic Characteristics of Clients at Participating Food Pantries *.

	Baseline		Mid-Point, After Phase 2		Post-Intervention, After Phase 3	
	Intervention (n = 34)	Comparison (n = 41)	Intervention (n = 33)	Comparison (n = 48)	Intervention (n = 34)	Comparison (n = 40)
Frequency of food pantry use, n (%)						
Once a week or more often	10 (29)	9 (22)	13 (38)	15 (31)	9 (26)	13 (28)
Twice a month	8 (24)	10 (24)	5 (15)	8 (17)	6 (18)	6 (13)
Once a month	9 (26)	17 (41)	14 (42)	22 (46)	14 (41)	22 (47)
Once every other month	2 (6)	0 (0)	0 (0)	1 (2)	1 (3)	4 (9)
Less often than once every other month	3 (9)	4 (10)	1 (3)	1 (2)	1 (3)	1 (2)
Other	2 (6)	1 (2)	0 (0)	1 (2)	2 (6)	1 (2)
Age, mean ± SD	60.86 ± 15.46 ^a^	53.37 ± 12.85 ^a^	62.82 ± 15.97 ^b^	49.48 ± 13.85 ^b^	66.47 ± 13.33 ^c^	55.27 ± 15.41 ^c^
Sex, % Female, n (%)	28 (82) ^d^	14 (34) ^d^	26 (79)	32 (67)	27 (79)	33 (70)
Race/ethnicity, n (%) Black/African American	32 (94)	36 (88)	31 (94)	37 (77)	33 (97)	41 (87)
Household size, mean ± SD	3.09 ± 1.48	2.66 ± 1.77	2.70 ± 1.36	3.63 ± 2.51	2.71 ± 1.45	2.64 ± 1.81
Number of children under age 18 in household, mean ± SD	0.91 ± 1.1	0.93 ± 1.54	0.70 ± 1.26	1.46 ± 2.14	0.82 ± 1.42	1.0 ± 1.44
SNAP recipients, n (%)	17 (50)	26 (63)	15 (46) ^e^	37 (77) ^e^	11 (32)	32 (68)
WIC recipients, n (%)	4 (12)	1 (2)	5 (15)	7 (15)	3 (9)	4 (9)
Employment status, n (%)						
Employed 30+ h/wk	8 (24) ^f^	0 (0) ^f^	3 (9)	1 (2)	2 (6)	1 (2)
Employed <30 h/wk	4 (12)	0 (0)	1 (3)	3 (6)	1 (3)	1 (2)
Unemployed	4 (12)	11 (27)	1(3) ^g^	30 (63) ^g^	11 (32)	22 (47)
Retired	11 (32)	9 (22)	19 (58) ^h^	5 (10) ^h^	15 (44)	2 (4)
Disabled	7 (21) ^i^	20 (49) ^i^	9 (27)	9 (19)	4 (12)	19 (40)
Other	0 (0)	0 (0)	0 (0)	0 (0)	0 (0)	1 (2)

* Statistically significant relationships with *p* < 0.05 are denoted with ^a–i^.

**Table 4:** Healthfulness of Client Selections at Intervention versus Comparison Food Pantries, measured by FAST Scores *.

	Intervention		Control	
	Median	Interquartile Range	Median	Interquartile Range
Baseline	57.84 ^a,e,f^	11.13	69.72 ^a,c^	8.98
	(n = 34)		(n = 41)	
Mid-Point–After Phase 2	66.32 ^e^	10.39	67.26 ^d^	12.06
	(n = 33)		(n = 48)	
Post-Intervention–After Phase 3	67.57 ^b,f^	22.78	58.36 ^b,c,d^	14.24
	(n = 34)		(n = 40)	

* Statistically significant differences with *p* < 0.05 are denoted with ^a–f^.

## Data Availability

The data presented in this study are available on request from the corresponding author. The data are not publicly available due to confidentiality considerations.
